# Education as a predictor of antidepressant and anxiolytic medication use after bereavement: a population-based record linkage study

**DOI:** 10.1007/s11136-016-1440-1

**Published:** 2016-10-21

**Authors:** Aideen Maguire, John Moriarty, Dermot O’Reilly, Mark McCann

**Affiliations:** 1 Centre of Excellence for Public Health, Queen’s University Belfast, Belfast, UK; 2 Administrative Data Research Network, Queen’s University Belfast, Belfast, UK; 3 MRC/CSO Social and Public Health Sciences Unit, University of Glasgow, Glasgow, UK

**Keywords:** Education, Mental health, Bereavement, Suicide, Administrative data, Antidepressant

## Abstract

**Purpose:**

Educational attainment has been shown to be positively associated with mental health and a potential buffer to stressful events. One stressful life event likely to affect everyone in their lifetime is bereavement. This paper assesses the effect of educational attainment on mental health post-bereavement.

**Methods:**

By utilising large administrative datasets, linking Census returns to death records and prescribed medication data, we analysed the bereavement exposure of 208,332 individuals aged 25–74 years. Two-level multi-level logistic regression models were constructed to determine the likelihood of antidepressant medication use (a proxy of mental ill health) post-bereavement given level of educational attainment.

**Results:**

Individuals who are bereaved have greater antidepressant use than those who are not bereaved, with over a quarter (26.5 %) of those bereaved by suicide in receipt of antidepressant medication compared to just 12.4 % of those not bereaved. Within individuals bereaved by a sudden death, those with a university degree or higher qualifications are 73 % less likely to be in receipt of antidepressant medication compared to those with no qualifications, after full adjustment for demographic, socio-economic and area factors (OR 0.27, 95 % CI 0.09,0.75). Higher educational attainment and no qualifications have an equivalent effect for those bereaved by suicide.

**Conclusions:**

Education may protect against poor mental health, as measured by the use of antidepressant medication, post-bereavement, except in those bereaved by suicide. This is likely due to the improved cognitive, personal and psychological skills gained from time spent in education.

**Electronic supplementary material:**

The online version of this article (doi:10.1007/s11136-016-1440-1) contains supplementary material, which is available to authorized users.

## Introduction

Educational attainment has been shown to be positively associated with mental health [1, 2]. Higher levels of education are associated with higher self-reported quality of life and lower risk of depression, schizophrenia and anxiety-related disorders [1–3]. Three major theories have been hypothesised in the literature to explain this association. The first is that educational attainment is merely a proxy indicator of socio-economic status, which is known to be associated with mental health [4]. The second is that education is an indicator of the cognitive resources necessary for sense-making and resilience [5]. The third is the social capital account, whereby formal education is assumed to increase an individual’s personal and social resources, which has a positive impact on their mental health [6].

Some isolated studies have also shown that more educated people cope more comprehensively and experience more rapid recovery following acutely stressful events [7]. One stressful life event likely to affect every individual in their lifetime is bereavement [8]. Approximately 10 % of bereaved individuals will go on to develop complicated grief, a prolonged expression of grief symptoms which can lead to increased risk of physical illness, poorer quality of life, depression and suicidality [9, 10]. Risk factors for complicated grief are not often studied, and the effect of education on mental health following bereavement has produced conflicting results [11, 12]. In addition, most of the associated studies have focused only on subgroups of the population [13]. In a study of bereaved older adults, those with lower educational attainment were found to be at an increased risk of developing complicated grief [11]. However, in a study of caregivers after bereavement there was no association between education and risk of complicated grief [12]. A study on child mortality found that higher levels of educational attainment were associated with lower levels of parental distress in reaction to infant death [13]. But this was highly specific with a cohort of 127 parents all bereaved under similar circumstances. Understanding how education moderates the effect of bereavement on mental health is important in order to understand the mechanisms underlying this association. Additionally, certain individuals are more vulnerable in their grief than others, and identifying this group is a practice and research imperative, both because intervening inappropriately can cause harm and because of the need to target resources effectively to ensure the highest quality of life in the bereaved [14].

This study capitalises on a unique data linkage project allowing for a comprehensive analysis of grief reactions at a population-wide level given cause of death, a range of demographic factors and educational attainment. It is the largest study in the UK to date analysing the effect of education on bereavement experience. Levels of stress response may differ given cause of death, and so bereavement exposure is divided into those bereaved by an “expected” death, i.e., by an illness, those bereaved by a sudden death and those bereaved by suicide. The study aims to (i) assess the effect of educational attainment on poor mental health outcomes post-bereavement and (ii) to explore the mechanisms underlying this association given bereavement exposure in relation to two of the three major theories for the association between education and mental health (1) that educational attainment is merely a proxy indicator of socio-economic status and (2) that education is an indicator of the cognitive resources necessary for sense-making and resilience.

## Method

### Data sources

This was a record linkage study based on the analysis of Census data and death records linked to indicators of mental health and area-level measures of disadvantage and conurbation. Data from Census returns were collated from the Northern Ireland Longitudinal Study (NILS), a representative sample of approximately 28 % of the Northern Ireland (NI) population (estimated at 1.84 million in June 2014) with inclusion in the cohort determined by birthdate. Individuals born on one of the 104 anonymous, pre-designated birth days are identified in the nationwide, centralised healthcare registration system and their demographic information and unique health and care number linked to Census returns data. The cohort is described in full elsewhere [15]. At the time of this study, the NILS sample contained information on individual’s demographics, health and care number and data from the 2001 Census returns including information on socio-economic status, household composition and educational attainment. Census information for individuals who were co-resident with NILS members on the Census day is also available providing information on the household environment.

To determine whether an individual within the NILS was bereaved, death data, including cause of death, was obtained from the General Register’s Office for all deaths in NI from April 2001 (Census day) to December 2009. This was linked to the dataset using a mixture of exact and probabilistic matching using name and date of birth fields. This allowed for the identification of deaths of co-resident household members. The data are described in detail elsewhere [16].

Psychotropic medication utilisation was used as a proxy indicator of mental health. Data relating to dispensed medications were extracted from the Enhanced Prescribing Database (EPD), a centralised collation of all prescriptions dispensed in community pharmacies in NI [17]. NI has a universal free healthcare system under the National Health Service (NHS), and all individuals are entitled to free-at-the-point-of-service health and social care. Information was extracted for all antidepressant and anxiolytic medications (British National Formulary (BNF) category 4.3 and 4.1.2), which have been used as proxy indicators for poor mental health in previous studies and have been shown to be predominantly prescribed for depression and anxiety disorders [18, 19]. While medication use is not a measure of well-being, it can be assumed that it is a good proxy for the presence of mental distress/disorder which is known to be highly correlated with well-being and associated with decreased quality of life [20, 21]. The information available from the prescription included: individual’s unique health and care number, general practice (GP) identifier, drug name and BNF category. The BNF is a standard reference used in the UK [22]. This was then linked to the NILS member dataset using one-to-one linkage of unique health and care numbers. All record linkages were carried out by the NILS data custodians. The final, and entirely anonymised, version of the data was housed within the secure environment of Northern Ireland Statistics and Research Agency (NISRA). The Office for Research Ethics Committees Northern Ireland approved the study (Ref: 06108/05/2013).

### Cohort description

The resulting dataset contained 2001 Census returns for 450,828 NILS members and their 818,670 co-residents, along with information on date and cause of death for any co-resident household member between 2001 and 2009 and the antidepressant or anxiolytic medication usage of any NILS member in January/February 2010. Analysis was restricted to 208,332 NILS members alive and resident in Northern Ireland in 2010, who had been living with at least one other co-resident and were aged between 25 and 74 years at the time of the 2001 Census. This age range allowed for a more accurate identification of educational attainment. The majority, 95.8 %, of co-residents were immediate family members (data not shown). Demographic, household and socio-economic indicators known to be associated with poor mental health were identified including gender [23], age [23], marital status [24], physical illness [25], socio-economic status [4], area-level deprivation [26] and rurality [27].

### Education

Educational attainment was measured using the response to the highest educational achievement question on the 2001 Census returns identifying those with no formal qualifications; those with a foundation certificate (usually for vocational training); those with 5+ GCSEs (standard qualifications attained at age 16 years); those with 2+ A levels (standard qualifications attained at age 18 years); or those with a university degree or higher.

### Individual characteristics

Gender was defined as male/female and age grouped into five 10-year age bands (25–34, 35–44, 45–54, 55–64 and 65–74 years). Marital status was divided into five groups: never married, married, co-habiting, separated or divorced and widowed. Limiting long-term illness was identified as a binary variable (yes/no) and used as an indicator of physical illness [28]. Socio-economic status was defined using a combination of standardised national NSSEC indicators (never worked/unemployed; routine/semi-routine; lower technical; own business; professional; student; economically inactive) as well as indicators of housing tenure and car ownership, which have been used in previous studies as indicators of economic resources [29].

### Area-level characteristics

A measure of disadvantage was extracted from the income deprivation domain of the Northern Ireland Multiple Deprivation Measure (NIMDM) which provides information on the proportion of the population in each area living in households in receipt of income-related benefits and tax credits in 2008/2009. Scores were ranked and split into quintiles containing approximately equal proportions of the population identifying affluent through to deprived areas. As there is no universally agreed definition of what constitutes an “urban” or “rural” area, for the purposes of this study level of conurbation was divided into three categories with settlements of ≥75,000 people classified as urban, settlements of 1000–75,000 people classified as intermediate and settlements with less than 1000 people classified as rural. The urban group encompasses NI’s two largest cities which are home to almost half the population.

### Bereavement

Bereavement was identified as the death of any co-resident household member between 2001 and 2009, identifying 4 discrete exposure categories: individuals who suffered no bereavement over the study period, those bereaved through illness, those bereaved by a sudden death (such as road traffic accidents or homicide) and those bereaved by suicide. These classifications were based on the appropriate ICD 10 codes (available on request).

### Mental health

Probable mental health condition was identified via receipt of antidepressant medication. For this study, we looked cross-sectionally at prescribing in January/February 2010 and individuals were classed as having poor mental health (MH) if they received at least one prescription for an antidepressant in either month and in good mental health if they did not receive a prescription. Most individuals receive prescriptions for antidepressants on a monthly basis, but using 2 months of prescribing data allows for the accurate capturing of missed scripts. Receipt of anxiolytic medication was also recorded as an additional proxy for poor mental health for sensitivity analyses.

### Analytical strategy

Analysis was divided into two stages. The first stage was descriptive analysis of the cohort to determine their demographic characteristics including educational attainment, bereavement status and mental health. Chi-squared tests were carried out to determine whether the bereaved and non-bereaved populations were significantly different. In the second stage, multi-level logistic regression models were constructed to determine whether educational attainment was independently associated with antidepressant use over and above bereavement status. The multi-level models adjusted for the clustering of individuals within GP practices, adjusting for any variation in prescribing practice. Demographic, household and socio-economic indicators known to be associated with poor MH were added to the model in an attempt to explain the association. Likelihood ratio (LR) tests were applied to determine whether there was an interaction between level of educational attainment and bereavement status. Sensitivity analyses were carried out identifying anxiolytic medication uptake in January/February 2010 as the outcome instead of antidepressant medication, as anxiolytic medication is sometimes used for the treatment of depression or depression symptoms, yielding similar results. In addition, bereavement was measured over the full study period due to the small numbers of those bereaved by suicide, but the effect of “time since bereavement” was tested by identifying a recently bereaved cohort, i.e., only those bereaved in the final 3 years of the study period closest to the outcome measurement. All analyses were carried out in Stata 13.

## Results

The study cohort consisted of 208,332 individuals from 355 different GP practices, aged between 25 and 74 years at the time of the 2001 Census, alive and resident in NI in January 2010 (mean age 45.1 years). As shown in Table 1, the majority of the cohort were female (52.8 %), married (74.1 %), with no or low-level educational qualifications (only 16.9 % with a degree or higher), employed (94.1 %), in good physical health (79.4 %) with car access and owned their own home (89.4 % with at least one car and 83.0 % own their own home). The cohort was evenly distributed across areas of deprivation with a higher proportion living in urban compared to rural areas.

**Table 1:** Demographic characteristics of the study cohort (*n* = 208,332) including bereavement status and proportion in receipt of antidepressant medication

Characteristic	Group size	% Cohort	% Bereaved relative	% on AD	*p*
*Gender*					
Male	98,313	47.2	7.3	8.3	*
Female	110,019	52.8	8.6	16.7	*
*Age group (at 2001 census)*					
25–34 years	51,883	24.9	5.1	10.9	*
35–44 years	57,614	27.7	5.1	13.6	*
45–54 years	46,182	22.2	7.2	14.1	*
55–64 years	33,899	16.3	11.0	12.5	*
65–74 years	18,754	9.0	21.1	12.2	*
*Marital status*					
Single	27,462	13.2	19.9	11.8	*
Married	154,366	74.1	6.4	11.9	*
Co-habiting	9899	4.8	3.2	12.4	*
Separated/divorced	12,033	5.8	5.8	23.7	*
Widowed	4572	2.2	4.4	16.5	*
*Limiting long-term illness*					
No	165,337	79.4	7.1	9.7	*
Yes	42,995	20.6	11.4	24.2	*
*Education*					
No qualifications	93,064	44.7	10.9	15.9	*
Foundation	36,735	17.6	5.4	12.7	*
5+ GSCE	30,040	14.4	6.3	10.7	*
A levels	13,269	6.4	5.0	10.0	*
Degree or higher	35,224	16.9	5.4	7.2	*
*NSSEC*					
Never work/unemployed	4931	2.4	13.3	14.0	*
Routine/semi-routine	68,946	33.1	9.3	16.5	*
Lower technical	19,130	9.2	7.8	11.9	*
Own business	13,452	6.4	7.5	8.3	*
Professional	94,614	45.4	6.5	9.9	*
Student	871	0.4	6.2	11.3	*
Economically inactive	6415	3.1	13.5	24.7	*
*Car access*					
No car	22,056	10.6	12.6	21.6	*
1 car	87,933	42.2	8.7	14.3	*
≥2 cars	98,343	47.2	6.3	9.4	*
*House tenure*					
Own house	172,955	83.0	7.6	11.1	*
Renting	35,377	17.0	10.0	20.9	*
*Urban/rural*					
Urban	78,384	37.6	7.9	13.9	*
Intermediate	70,032	33.8	7.2	13.5	*
Rural	59,646	28.6	9.0	9.2	*
*Area-level deprivation*					
Least deprived	42,575	20.4	5.9	9.2	*
2	42,655	20.5	7.0	11.2	*
3	40,050	19.2	8.0	12.4	*
4	38,549	18.5	9.1	14.4	*
Most deprived	33,579	16.1	9.9	18.3	*
Missing	10,924	5.2	9.9	11.1	*
*Bereavement status*					
No bereavement	191,720	92.0	0	12.4	*
Bereaved by illness/other	15,812	67.6	95.2	15.7	*
Bereaved by sudden death	487	0.2	2.9	22.4	*
Bereaved by suicide	313	0.2	1.9	26.5	*

*AD* antidepressant medication

* *p* *<* 0.001 for *χ* ^2^ tests to determine whether each characteristic differs between the bereaved population and the whole population cohort

### Bereavement exposure and antidepressant use

Bereavement affected 8.0 % of the cohort over the study period with a larger proportion of older people bereaved compared to younger. The vast majority of co-residents who died were family members with over 78 % of those bereaved losing a spouse, parent or child (data not shown). Educational background was also associated with bereavement experience, with a higher proportion of those with no qualifications bereaved (10.9 %) compared to those with a degree or higher (5.4 %). The majority of bereaved individuals were bereaved through an illness with only 2.9 % of the bereaved cohort bereaved by a sudden death and 1.9 % bereaved by suicide (see bottom of Table 1).

Prescribing was highest amongst those with no qualifications with 15.9 % receiving an antidepressant in January/February 2010 compared to just 7.2 % of those with a degree or higher. Prevalence of antidepressant uptake also increased linearly across the four categories of bereavement with only 12.4 % of those who were not bereaved receiving an antidepressant in January/February 2010 compared to 15.7 % of those bereaved by illness, 22.4 % of those bereaved by a sudden death and 26.5 % of those bereaved by suicide.

### Likelihood of antidepressant use given educational attainment

Table 2 shows the expected relationship between educational attainment and poor MH as measured by receipt of antidepressant medication. Even after full adjustment for gender, age, marital status, physical illness, socio-economic status, conurbation, area-level deprivation and bereavement status, education had an independent effect on the likelihood of receipt of antidepressant medication. In the fully adjusted model, individuals with a degree or higher were 38 % less likely to receive antidepressant medication compared to those with no qualifications (OR 0.62, 95 % CI 0.58, 0.65).

**Table 2:** Multi-level models calculating the likelihood of receiving antidepressant medication in January/February 2010 given educational attainment, adjusting for clustering of individuals by GP Practice

	Model 1	Model 2	Model 3	Model 4	Model 5
	Unadjusted	+Adj sex, age, marital status and illness	+Adj SES (NSSEC, tenure and car access)	+Adj conurbation and area-level deprivation	+Adj bereavement status
Highest educational qualification					
None	1.00	1.00	1.00	1.00	1.00
Foundation	0.78 (0.75, 0.80)	0.78 (0.75, 0.81)	0.86 (0.82, 0.89)	0.86 (0.83, 0.90)	0.86 (0.83, 0.90)
5+GCSE	0.65 (0.63, 0.68)	0.66 (0.64, 0.69)	0.77 (0.73, 0.80)	0.77 (0.73, 0.81)	0.77 (0.74, 0.81)
A levels	0.60 (0.57, 0.64)	0.63 (0.60, 0.68)	0.74 (0.70, 0.79)	0.74 (0.70, 0.79)	0.75 (0.70, 0.80)
Degree+	0.42 (0.40,0.44)	0.50 (0.48,0.53)	0.61 (0.58,0.64)	0.61 (0.58,0.65)	0.62 (0.58,0.65)
Gender					
Male		1.00	1.00	1.00	1.00
Female		2.21 (2.15,2.28)	2.12 (2.06,2.18)	2.12 (2.06,2.19)	2.11 (2.05,2.18)
Age (at 2001 census)					
25–34 years		1.00	1.00	1.00	1.00
35–44 years		1.12 (1.08,1.17)	1.16 (1.11,1.20)	1.16 (1.12,1.21)	1.15 (1.11,1.20)
45–54 years		1.05 (1.00,1.10)	1.13 (1.08,1.18)	1.13 (1.09,1.18)	1.12 (1.07,1.17)
55–64 years		0.73 (0.70,0.77)	0.79 (0.75,0.82)	0.79 (0.75,0.83)	0.77 (0.73,0.81)
65–74 years		0.63 (0.60,0.67)	0.66 (0.62,0.70)	0.67 (0.63,0.71)	0.64 (0.60,0.68)
Marital status					
Married		1.00	1.00	1.00	1.00
Single		1.00 (0.96,1.04)	0.90 (0.86,0.94)	0.90 (0.86,0.94)	0.87 (0.83,0.91)
Co-habiting		1.12 (1.05,1.20)	1.03 (0.97,1.10)	1.02 (0.96,1.09)	1.02 (0.95,1.09)
Separated/divorced		1.56 (1.49,1.64)	1.29 (1.23,1.36)	1.28 (1.22,1.35)	1.28 (1.22,1.35)
Widowed		1.02 (0.94,1.11)	0.96 (0.88,1.04)	0.95 (0.88,1.04)	0.97 (0.90,1.06)
Limiting long-term illness					
No		1.00	1.00	1.00	1.00
Yes		2.84 (2.76,2.94)	2.68 (2.60,2.77)	2.67 (2.59,2.76)	2.67 (2.59,2.76)
NSSEC					
Professional			1.00	1.00	1.00
Own business			0.89 (0.84,0.96)	0.91 (0.85,0.98)	0.91 (0.85,0.98)
Lower technical routine/semi-routine			1.06 (1.01,1.12)	1.05 (0.99,1.10)	1.05 (0.99,1.10)
Never work/unemployed			1.11 (1.08,1.15)	1.10 (1.06,1.14)	1.10 (1.06,1.14)
Student			1.00 (0.92,1.10)	1.00 (0.92,1.09)	1.00 (0.91,1.09)
Economically			0.98 (0.79,1.22)	0.98 (0.79,1.22)	0.99 (0.79,1.23)
Inactive			1.17 (1.10,1.26)	1.18 (1.10,1.26)	1.17 (1.09,1.25)
Car access					
≥2 Cars			1.00	1.00	1.00
1 Car			1.21 (1.18,1.25)	1.17 (1.13,1.23)	1.17 (1.13,1.21)
No car			1.34 (1.28,1.41)	1.26 (1.20,1.33)	1.26 (1.19,1.32)
House tenure					
Own house			1.00	1.00	1.00
Renting			1.32 (1.27,1.37)	1.29 (1.24,1.34)	1.29 (1.24,1.34)
Urban/rural					
Rural				1.00	1.00
Intermediate				1.18 (1.14,1.23)	1.19 (1.14,1.24)
Urban				1.18 (1.12,1.24)	1.18 (1.12,1.24)
Deprivation					
Least deprived				1.00	1.00
2				1.13 (1.08,1.19)	1.13 (1.07,1.18)
3				1.13 (1.08,1.19)	1.13 (1.08,1.19)
4				1.17 (1.11,1.23)	1.16 (1.11,1.22)
Most deprived				1.23 (1.17,1.30)	1.23 (1.16,1.29)
Missing				1.16 (1.08,1.25)	1.16 (1.07,1.25)
Bereavement status					
No bereavement					1.00
Bereaved by illness/other					1.22 (1.16,1.29)
Bereaved sudden death					1.83 (1.46,2.29)
Bereaved by suicide					1.85 (1.85,2.41)
*χ* ^2^ (MLM vs. logistic)	1043.77	584.56	449.77	396.86	395.86
*p*	0.000	0.000	0.000	0.000	0.000
Variance	0.082	0.057	0.048	0.044	0.044
Variance partition coefficient	2.43	1.70	1.32	1.32	1.32

Figures show OR and 95 % CI (pop = 208 332; 355 GP practices)

Adjusting for bereavement status (model 5) does not appear to have a significant impact on the relationship between education and receipt of antidepressant medication (model 4). The significant Chi-squared test (MLM vs. logistic) across models illustrates that the multi-level model is a better fit to the data than a logistic model which does not account for GP variation. The variation partition coefficient illustrates the amount of variation attributable to GP Practice differences and in the fully adjusted model (Table 2, model 5) GP Practice variation accounts for only 1.32 % of the variation in prescribing. There was, however, an interaction between bereavement status and level of education on likelihood of antidepressant medication (LR Test = 6.88, *p* = 0.0087, *df* = 34). To further explore this, the models were stratified by bereavement category (Table 3).

**Table 3:** Multi-level models calculating the likelihood of receiving antidepressant medication in January/February 2010 given educational attainment stratified by bereavement status

	% Population on antidepressants		Model 1	Model 2	Model 3	Model 4
			Unadjusted	+Adj sex, age, marital status and illness	+Adj SES (NSSEC, tenure and car)	+Adj conurbation and area deprivation
No bereavement (*n* = 191,720)	12.4	*Educational attainment*				
		No qualifications	1.00	1.00	1.00	1.00
		Foundation level	0.78 (0.75, 0.81)	0.78 (0.75, 0.81)	0.85 (0.82, 0.89)	0.86 (0.82, 0.89)
		5+ GSCE	0.65 (0.63, 0.68)	0.66 (0.63, 0.69)	0.76 (0.72, 0.80)	0.76 (0.73, 0.80)
		Secondary level (A levels)	0.61 (0.57, 0.65)	0.64 (0.60, 0.68)	0.74 (0.69, 0.79)	0.75 (0.70, 0.80)
		Third level (degree+)	0.42 (0.40,0.44)	0.50 (0.47, 0.52)	0.60 (0.57, 0.64)	0.61 (0.58, 0.64)
Bereaved by illness/other (*n* = 15,812)	15.7	*Educational attainment*				
		No qualifications	1.00	1.00	1.00	1.00
		Foundation level	0.81 (0.71, 0.94)	0.89 (0.76, 1.03)	0.96 (0.82, 1.12)	0.95 (0.81, 1.10)
		5+ GSCE	0.71 (0.61, 0.82)	0.76 (0.65, 0.89)	0.87 (0.74, 1.02)	0.86 (0.73, 1.02)
		Secondary level (A levels)	0.57 (0.44, 0.73)	0.65 (0.50, 0.85)	0.74 (0.56, 0.97)	0.73 (0.55, 0.96)
		Third level (degree+)	0.51 (0.44, 0.60)	0.62 (0.53, 0.74)	0.74 (0.61, 0.89)	0.73 (0.61, 0.88)
Bereaved by sudden death (*n* = 487)	22.4	*Educational attainment*				
		No qualifications	1.00	1.00	1.00	1.00
		Foundation level	0.49 (0.24, 1.00)	0.46 (0.21, 1.01)	0.45 (0.19, 1.03)	0.41 (0.18, 0.96)
		5+ GSCE	0.40 (0.18, 0.89)	0.38 (0.17, 0.86)	0.38 (0.15, 0.93)	0.37 (0.15, 0.92)
		Secondary level (A levels)	0.57 (0.19, 1.75)	0.44 (0.13, 1.47)	0.40 (0.11, 1.44)	0.36 (0.10, 1.31)
		Third level (degree+)	0.33 (0.14, 0.80)	0.32 (0.13, 0.82)	0.29 (0.11, 0.82)	0.27 (0.09, 0.75)
Bereaved by suicide (*n* = 313)	26.5	*Educational attainment*				
		No qualifications	1.00	1.00	1.00	1.00
		Foundation level	0.91 (0.45, 1.85)	1.16 (0.52, 2.62)	1.26 (0.50, 3.15)	1.26 (0.48, 3.32)
		5+ GSCE	0.58 (0.21, 1.67)	0.67 (0.22, 2.02)	0.85 (0.23, 3.14)	0.86 (0.22, 3.37)
		Secondary level (A levels)	0.86 (0.26, 2.81)	0.79 (0.22, 2.78)	1.07 (0.24, 4.83)	0.96 (0.21, 4.50)
		Third level (degree+)	0.49 (0.14, 1.70)	0.86 (0.23, 3.15)	0.98 (0.21, 4.54)	1.42 (0.29, 7.00)

Adjusting for clustering of individuals by GP Practice. Figures show OR and 95 % CI (pop = 208 332; 355 GP practices)

### Likelihood of antidepressant use post-bereavement given educational attainment

**Fig. 1:**
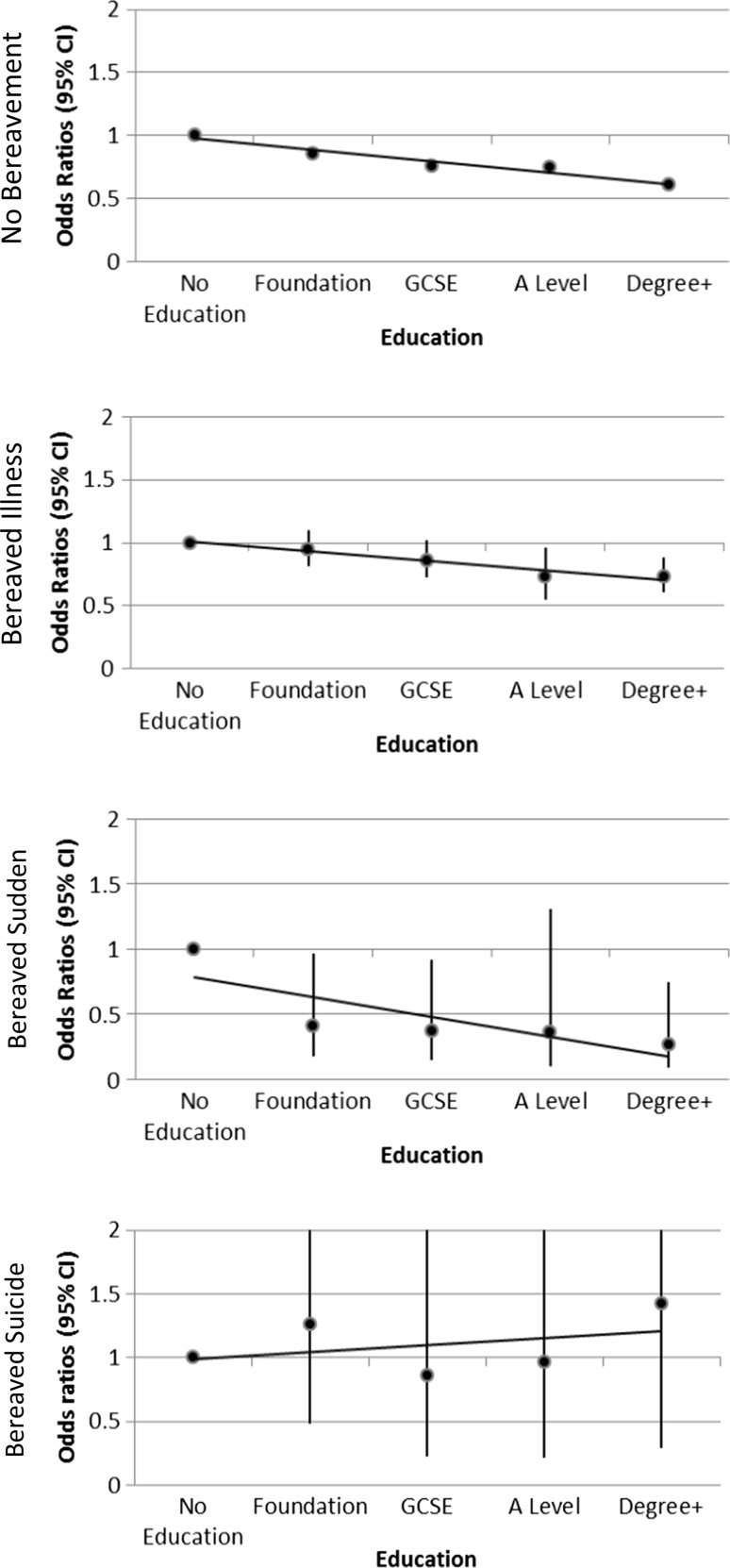
Likelihood of poor mental health post-bereavement by educational attainment for each bereavement exposure fully adjusted for sex, age, marital status, illness, socio-economic status, conurbation and area deprivation

Separate multi-level logistic regression models were constructed again adjusting for the clustering of individuals within GP Practices. In the unadjusted model, in the subgroups that suffered no bereavement or were bereaved by illness (Table 3, model 1) there was a clear stepwise decrease in the likelihood of antidepressant medication as level of educational attainment increased. After full adjustment (Table 3, model 4) this association was attenuated, but remained. In the subgroup who were not bereaved, individuals with a degree or higher were 39 % less likely to receive antidepressant medication compared to those with no qualifications after full adjustment for demographic, socio-economic and area-level factors (OR 0.61, 95 % CI 0.58, 0.64). In the subgroup who suffered bereavement through illness, individuals with a degree or higher were 27 % less likely to receive antidepressant medication after full adjustment for confounding factors (OR 0.73, 95 % CI 0.61, 0.88). The protective effect of education was much more evident for individuals bereaved by a sudden death. Those bereaved by a sudden death with a degree or higher were 73 % less likely to receive antidepressant medication compared to those bereaved by a sudden death with no qualifications (OR 0.27, 95 % CI 0.09, 0.75), in the fully adjusted model (Table 3, model 4). However, in the subgroup who were bereaved by suicide the association between education and antidepressant medication loses significance. Individuals with a degree or higher are not at an increased or decreased likelihood of receipt of antidepressant medication post-bereavement by suicide compared to those individuals with no qualifications (OR 1.42, 95 % CI 0.29, 7.00). This is shown diagrammatically in Fig. 1.

### Sensitivity analyses

Using receipt of anxiolytic medication in place of antidepressant medication yields similar results (see online resource Tables 2a and 3a). In the fully adjusted model in Table 2a, individuals with a degree or higher were 43 % less likely to receive anxiolytic medication compared to those with no qualifications (OR 0.57, 95 % CI 0.51, 0.62). After stratification by bereavement status, in the fully adjusted models for subgroups that suffered no bereavement (Table 3a, model 4) there was a clear stepwise decrease in the likelihood of anxiolytic medication as level of educational attainment increased. However, this association was not present in those bereaved by suicide. Time since bereavement was tested using analysis limiting the identification of “bereavement” as “bereaved in the 3 years prior to prescribing” showing similar trends to the overall analysis (see online resource Table 4), with those with higher educational attainment less likely to receive antidepressant medication when not bereaved or bereaved by an illness, and the association not significant for those bereaved by suicide. However, due to very small numbers the results of these sensitivity analyses yielded very large confidence intervals and cannot be trusted.

## Discussion

This is the largest study of its kind to look at the effect of education on mental health post-bereavement in a representative sample of the population. Firstly, the study illustrates that more bereaved individuals are receiving antidepressant medication compared to those not bereaved, 12.4 % of the non-bereaved population on antidepressant medication compared to 26.5 % of those bereaved by suicide. As expected, higher educational attainment has a protective effect on the likelihood of receipt of antidepressant medication, even after full adjustment for demographic and socio-economic factors known to be associated with mental health. However, when observing mental health in those who have experienced bereavement, the protective effect of educational attainment varies depending on the circumstance of bereavement. This is clearly illustrated in Fig. 1. Increasing education is associated with a decreased likelihood of antidepressant medication for all individuals (bereaved or non-bereaved), except those bereaved by suicide. These findings may shed light on the mechanisms underlying the association between education and mental health in general.

### Comparison with other studies

The observed association between educational attainment and general mental health supports decades of previous work that higher education is associated with better mental health. Two of the three major theories stating that (i) educational attainment is merely a proxy indicator of socio-economic status and (ii) education is an indicator of the cognitive resources necessary for sense-making and resilience can be explored in more detail from the results [4, 5].

This study illustrates that education has a protective effect against the risk of antidepressant medication in the non-bereaved population and in the population bereaved by illness. Adjusting for socio-economic status does not alter this association. Therefore, education is not merely a proxy indicator of socio-economic status. However, socio-economic inequality accounts for some of the observed variation in mental health across levels of educational attainment with associations attenuated substantially after the addition of the socio-economic variables into the model. Educational attainment will identify individuals from more affluent backgrounds as they are more likely to complete education up to the age of 18 years, compared to those from less affluent backgrounds [30]. Also, lower levels of educational attainment lower the possibly for employment, especially in professional jobs, and so can result in a lower socio-economic position or unemployment, which in turn can affect mental health [31]. Therefore, while targeting mental health interventions to those in more deprived circumstances remains important, the residual effect of education status suggests that additional targeting of services to bereaved persons with low educational attainment is also necessary.

Education also protects against risk of poor MH after a sudden death. Coping with a sudden death requires a degree of sense-making and personal resilience which may be attributable to level of educational attainment [32]. Education has been proposed as a resource which improves cognitive ability and makes individuals better at “self-management” when problems occur [33]. The ability to rationalise death and understand its expectedness as part of the life cycle leads to a greater assimilation of bereavement and a less negative effect on mental health. A sudden death, though traumatic, could be more easily rationalised by someone of greater cognitive ability and have a greater negative effect on those with lower cognitive skills. Education also improves social networks which can buffer the ill effects of stressful life events [34].

Education, however, does not protect against poor MH in those bereaved by suicide. This conflicting trend can be observed easily in Fig. 1. Suicide bereavement though is distinct from other bereavements in three significant ways: the thematic content of the grief, the social processes surrounding the survivor, and the impact suicide being on family systems [35]. In these circumstances, the grieving process may be hindered by factors such as delays in funeral arrangements due to coroner’s investigations, barriers to support due to the ongoing stigma surrounding suicide and feelings of guilt or a feeling that the death was preventable [32]. It can be argued that those with a higher educational attainment and hence more personal and social resources should have better mental health outcomes in all cases of bereavement, including suicide. However, the societal stigma attached to suicide may cause individuals to be too ashamed to use their usual support networks and agents of social support to be less forthcoming than in other cases of bereavement. It may also be less easy to rationalise a death which is perceived to be preventable. It is not possible to ascertain through this study if social capital or cognitive ability plays a greater role in explaining the association between education and mental health.

### Potential limitations

For the interpretation of observed trends, we assumed that antidepressant prescription is a fair proxy for population-level mental health. However, this measure does not account for individual differences in help-seeking behaviour or GP attendance and does not capture those who are referred to alternative services such as counselling. The data will therefore not capture all those who have poor mental health post-bereavement. Nevertheless, if it were the case that antidepressant prescription reflected greater likelihood of help-seeking behaviour, we would expect higher uptake of antidepressant prescription with higher educational attainment as previous studies have shown greater help-seeking behaviour in groups with higher educational attainment [36]. Given that the trend is the reverse, this seems unlikely to have distorted the results. It is possible that more educated individuals may be less willing to seek treatment via prescription medication and more willing to avail of alternative therapy [37]; however, in a review of predictors of psychotropic medication use low educational attainment was associated with increased use in only 3 out of 27 studies [38]. As evidence suggests that many of those suffering from complicated or prolonged grief do not access mental health services and are more likely to only be seen by primary care physicians, it can be assumed that pharmacological therapy is a likely treatment route, and antidepressant medication has been shown to be an effective treatment option [39–41]. Although antidepressant medication is sometimes prescribed for indications other than depression or anxiety, evidence exists indicating the substantial correlation between antidepressant medication and depression diagnosis, suggesting that this is an acceptable proxy measure of mental distress/disorder which is known to be associated with decreased quality of life [18, 19, 21].

Personal and family history of mental health may also play a role in mental health post-bereavement [42]. Pre-bereavement general health was included in the models in an attempt to make some adjustment for this, but further studies are needed to compliment these finding which can take in consideration family history of mental illness. The analysis has all the limitations associated with a cross-sectional study. Due to the newness of the enhanced prescribing database and the delay in the recording of death data, the sample of concurrent bereavement and prescription data was too small to carry out the subgroup analysis executed above. Sensitivity analysis breaking up the exposure period to more recent and more long-term bereavements was carried out, but due to the rarity of the event of death by suicide, meaningful analysis was not possible (see online resource Table 4). As the enhanced prescribing database develops and with the addition of more up-to-date death data, more accurate analysis on the temporal ordering of prescribing instances after bereavement can be carried out to further understand the association between level of educational attainment and mental health.

While bereavement is defined in relation to a death occurring within the household, it is the case that some “significant others” will live outside the household and therefore will not contribute to the bereavement variable. However, this potential misclassification bias does not alter the message of the paper, but only affects specificity. The bereaved variable accurately captures deaths of co-residents.

## Conclusion

The implications of these study findings are twofold. First, the study illustrates that education may protect against poor mental health outcomes post-bereavement, except for those bereaved by suicide. This is likely due to the improved cognitive, personal and psychological skills gained from time spent in education. Socio-economic status and deprivation explain some of the relationship between poor MH and bereavement outcomes, and so targeted interventions aimed at those from lower socio-economic backgrounds are justified. However, special attention should be paid to all individuals bereaved by suicide irrespective of socio-demographic factors. There is a need for more work aimed at understanding reaction to suicide bereavement and the mechanisms for a positive mental health outcome after this event. Secondly, the study sheds new light on the mechanisms underlying the association between education and mental health. The association cannot fully be explained by the theory that education is merely an indicator of socio-economic status, and so education must improve personal resources. A more in-depth analysis of these associations is warranted.

## Electronic supplementary material

Below is the link to the electronic supplementary material.

Supplementary material 1 (DOCX 26 kb)
