# Supplementary material for: Education as a predictor of antidepressant and anxiolytic medication use after bereavement: a population-based record linkage study
Source: Qual Life Res. 2016 Oct 21;26(5):1251–62. doi: 10.1007/s11136-016-1440-1 (PMC5376389; doi:10.1007/s11136-016-1440-1)
Supplement: Supplementary file 1 — Supplementary material 1 (DOCX 26 kb) [file 11136_2016_1440_MOESM1_ESM.docx]

**Table 2a: Multi-Level Models calculating the likelihood of receiving anxiolytic medication in Jan/Feb 2010 given educational attainment, adjusting for clustering of individuals by GP Practice. Figures show OR and 95 % CI (pop=208 332; 355 GP practices)**

|  |  | **Model 1** | **Model 2** | **Model 3** | **Model 4** | **Model 5** |
| --- | --- | --- | --- | --- | --- | --- |
|  |  | **Unadjusted** | **+adj**  **Sex, age, Marital**  **Status & illness** | **+adj**  **SES (NSSEC, tenure**  **& car access)** | **+adj**  **Conurbation & Area Deprivation** | **+adj**  **Bereavement**  **Status** |
| **Highest Educational Qualification** | **None**  **Foundation**  **5+ GCSE**  **A Levels**  **Degree+** | 1.00  0.58 (0.55,0.62)  0.46 (0.43,0.50) 0.40 (0.36,0.45) 0.28 (0.26,0.31) | 1.00  0.72 (0.69,0.77)  0.56 (0.51,0.60) 0.52 (0.46,0.58) 0.41 (0.38,0.45) | 1.00  0.84 (0.78,0.90)  0.70 (0.64,0.76) 0.67 (0.59,0.75) 0.56 (0.50,0.61) | 1.00  0.84 (0.79,0.90)  0.71 (0.65,0.77) 0.67 (0.59,0.76) 0.56 (0.51,0.62) | 1.00  0.85 (0.79,0.91)  0.71 (0.65,0.77) 0.67 (0.60,0.76) 0.57 (0.51,0.62) |
| **Gender** | **Male**  **Female** |  | 1.00  2.14 (2.05,2.25) | 1.00  2.00 (1.90,2.10) | 1.00  2.00 (1.90,2.10) | 1.00  1.99 (1.89,2.10) |
| **Age**  **(at 2001 Census)** | **25-34 years**  **35-44 years**  **45-54 years**  **55-64 years**  **65-74 years** |  | 1.00  1.20 (1.11,1.29)  1.24 (1.15,1.34)  1.19 (1.10,1.30)  1.05 (0.96,1.16) | 1.00  1.25 (1.17,1.34)  1.38 (1.28,1.49)  1.36 (1.25,1.48)  1.17 (1.06,1.29) | 1.00  1.25 (1.17,1.35)  1.39 (1.28,1.50)  1.37 (1.26,1.49)  1.19 (1.08,1.31) | 1.00  1.24 (1.16,1.34)  1.37 (1.27,1.48)  1.34 (1.23,1.46)  1.15 (1.04,1.27) |
| **Marital Status** | **Married**  **Single**  **Co-habiting**  **Separated/Divorced**  **Widowed** |  | 1.00 1.19 (1.11,1.28) 1.27 (1.14,1.42) 1.78 (1.65,1.91) 1.17 (1.03,1.32) | 1.00 0.98 (0.90,1.05) 1.12 (1.00,1.25) 1.35 (1.25,1.46) 1.04 (0.92,1.17) | 1.00 0.97 (0.90,1.05) 1.10 (0.99,1.23) 1.33 (1.23,1.44) 1.03 (0.92,1.17) | 1.00 0.95 (0.88,1.03) 1.10 (0.99,1.23) 1.34 (1.24,1.44) 1.05 (0.93,1.19) |
| **Limiting long-term illness** | **No**  **Yes** |  | 1.00  3.08 (2.94,3.24) | 1.00  2.78 (2.65,2.92) | 1.00  2.77 (2.64,2.91) | 1.00  2.77 (2.64,2.91) |
| **NSSEC** | **Professional**  **Own business**  **Lower technical**  **routine/semi-routine**  **Never work/unemployed**  **Student**  **Economically Inactive** |  |  | 1.00  0.93 (0.82,1.05)  1.03 (0.94,1.13)  1.14 (1.07,1.21)  1.03 (0.89,1.19)  0.90 (0.60, 1.37)  1.70 (1.54,1.87) | 1.00  0.95 (0.84,1.08)  1.01 (0.93,1.11)  1.12 (1.05,1.19)  1.02 (0.88,1.18)  0.91 (0.60, 1.37)  1.69 (1.53,1.87) | 1.00  0.95 (0.84,1.08)  1.01 (0.93,1.11)  1.12 (1.05,1.19)  1.01 (0.88,1.17)  0.91 (0.60, 1.38)  1.69 (1.53,1.86) |
| **Car Access** | **≥2 cars**  **1 car**  **No car** |  |  | 1.00  1.28 (1.21,1.35)  1.48 (1.37,1.60) | 1.00  1.22 (1.15,1.29)  1.36 (1.26,1.48) | 1.00  1.22 (1.15,1.29)  1.35 (1.25,1.47) |
| **House Tenure** | **Own House**  **Renting** |  |  | 1.00 1.49 (1.40,1.58) | 1.00 1.44 (1.36,1.52) | 1.00 1.44 (1.35,1.52) |
| **Urban/Rural** | **Rural**  **Intermediate**  **Urban** |  |  |  | 1.00  1.18 (1.10,1.26)  1.32 (1.21,1.44) | 1.00  1.18 (1.10,1.27)  1.33 (1.22,1.45) |
| **Deprivation** | **Least Deprived**  **2**  **3**  **4**  **Most deprived**  **Missing** |  |  |  | 1.00  1.14 (1.05,1.24)  1.16 (1.06,1.27)  1.22 (1.13,1.34)  1.34 (1.22,1.46)  1.17 (1.03,1.34) | 1.00  1.14 (1.05,1.24)  1.16 (1.06,1.26)  1.22 (1.12,1.33)  1.33 (1.22,1.46)  1.17 (1.02,1.33) |
| **Bereavement Status** | **No bereavement**  **Bereaved by illness/other**  **Bereaved Sudden Death**  **Bereaved by Suicide** |  |  |  |  | 1.00  1.14 (1.05,1.23)  1.56 (1.08,2.24)  2.07 (1.43,3.00) |
| **Chi2 (MLM vs. Logistic)** |  | 797.6 | 555.81 | 454.95 | 377.14 | 375.88 |
| **p** |  | 0.000 | 0.000 | 0.000 | 0.000 | 0.000 |
| **Variance** |  | 0.181 | 0.142 | 0.123 | 0.108 | 0.108 |
| **Variance Partition Coefficient** |  | 5.20 | 4.14 | 3.60 | 3.18 | 3.18 |

**Table 3a: Multi-Level Models calculating likelihood of receiving anxiolytic medication given educational attainment stratified by bereavement status. Adjusting for clustering of individuals by GP Practice. Figures show OR and 95% CI (pop=208 322; 355 GP practices)**

|  |  |  | **Model 1** | **Model 2** | **Model 3** | **Model 4** |
| --- | --- | --- | --- | --- | --- | --- |
|  | **% population on Anxiolytics** |  | **Unadjusted** | **+adj**  **Sex, age, Marital**  **Status & illness** | **+adj**  **SES (NSSEC, tenure**  **& car access)** | **+adj**  **Conurbation & Area Deprivation** |
| **Not Bereaved**  **(191,720)** | 3.9 | **Educational Attainment**  No Qualifications  Foundation/5+ GCSE  Secondary Level (A levels)  Third Level (Degree+) | 1.00  0.53 (0.51,0.56)  0.39 (0.35,0.45)  0.29 (0.26,0.31) | 1.00  0.64 (0.61,0.68)  0.51 (0.45,0.57)  0.41 (0.38,0.45) | 1.00  0.78 (0.73,0.84)  0.66 (0.58,0.75)  0.57 (0.51,0.63) | 1.00  0.79 (0.74,0.84)  0.66 (0.58,0.75)  0.57 (0.52,0.64) |
| **Bereaved Illness/other (15,812)** | 5.6 | **Educational Attainment**  No Qualifications  Foundation/5+ GCSE  Secondary Level (A levels)  Third Level (Degree+) | 1.00  0.58 (0.48,0.70)  0.59 (0.39,0.88)  0.33 (0.24,0.45) | 1.00  0.67 (0.55,0.82)  0.75 (0.50,1.14)  0.44 (0.32,0.61) | 1.00  0.82 (0.67,1.01)  0.94 (0.62,1.44)  0.59 (0.42,0.83) | 1.00  0.82 (0.66,1.01)  0.94 (0.62,1.43)  0.59 (0.42,0.84) |
| **Bereaved Sudden**  **(487)** | 7.0 | **Educational Attainment**  No Qualifications  Foundation/5+ GCSE  Secondary Level (A levels)  Third Level (Degree+) | 1.00  0.33 (0.11,0.96)  0.97 (0.22,4.39)  0.19 (0.02,1.41) | 1.00  0.35 (0.11,1.05)  0.94 (0.19,4.71)  0.19 (0.02,1.46) | 1.00  0.48 (0.14,1.68)  1.39 (0.22,8.84)  0.22 (0.02,2.06) | 1.00  0.51 (0.14,1.85)  1.54 (0.22,10.65)  0.27 (0.02,2.67) |
| **Bereaved Suicide**  **(313)** | 11.5 | **Educational Attainment**  No Qualifications  Foundation/5+ GCSE  Secondary Level (A levels)  Third Level (Degree+) | 1.00  0.44 (0.17,1.14)  0.27 (0.03,2.54)  0.51 (0.09,2.77) | 1.00  0.45 (0.21,1.24)  0.25 (0.19,2.81)  0.66 (0.09,4.22) | 1.00  0.54 (0.17,1.74)  0.31 (0.02,3.04)  1.08 (0.16,9.01) | 1.00  0.55 (0.17,1.76)  0.37 (0.03,3.89)  1.38 (0.19,9.97) |

*Note: as no individual within the “5+ GCSE” category bereaved by sudden death received anxiolytic medication we aggregated education into 4 categories for analysis using anxiolytic medication as the outcome*

**Table 4: Multi-Level Models calculating likelihood of receiving antidepressant medication given educational attainment stratified by bereavement status, for those recently bereaved (in last 3 years). Adjusting for clustering of individuals by GP Practice. Figures show OR and 95% CI (pop=208 322; 355 GP practices**

|  |  |  | **Model 1** | **Model 2** |
| --- | --- | --- | --- | --- |
|  | **% population on Antidepressants** |  | **Unadjusted** | **Fully adjusted*** |
| **Not Bereaved**  **(191,720)** | 12.4 | **Educational Attainment**  No Qualifications  Foundation Level  5+ GCSE  Secondary Level (A levels)  Third Level (Degree+) | 1.00  0.78 (0.75,0.81)  0.65 (0.63,0.68)  0.61 (0.57,0.65)  0.42 (0.40,0.44) | 1.00  0.86 (0.82,0.89)  0.77 (0.73,0.80)  0.75 (0.70,0.80)  0.61 (0.58,0.65) |
| **Bereaved Illness/other (6,064)** | 15.7 | **Educational Attainment**  No Qualifications  Foundation Level  5+ GCSE  Secondary Level (A levels)  Third Level (Degree+) | 1.00  0.80 (0.64,1.00)  0.64 (0.51,0.82)  0.48 (0.31,0.75)  0.59 (0.46,0.76) | 1.00  0.89 (0.69,1.14)  0.73 (0.56,0.95)  0.60 (0.38,0.96)  0.77 (0.58,1.02) |
| **Bereaved Sudden**  **(132)** | 22.4 | **Educational Attainment**  No Qualifications  Foundation Level  5+ GCSE  Secondary Level (A levels)  Third Level (Degree+) | 1.00  1.27 (0.33,4.93)  0.66 (0.16,2.73)  0.67 (0.04,11.01)  0.11 (0.01,1.45) | 1.00  1.67 (0.01,189.58)  0.94 (0.02, 47.71)  0.03 (0.01,65.54)  0.01 (0.01, 8.23) |
| **Bereaved Suicide**  **(102)** | 26.5 | **Educational Attainment**  No Qualifications  Foundation Level  5+ GCSE  Secondary Level (A levels)  Third Level (Degree+) | 1.00  0.22 (0.04,1.65)  0.38 (0.04,3.78)  0.63 (0.08,5.14)  0.85 (0.15,4.96) | 1.00  0.36 (0.01,2987)  0.43 (0.01,48145)  1.64 (0.04,62.02)  1.70 (0.06,51.8) |

**adjusted for sex, age, marital status, illness, NSSEC, car ownership, tenure, conurbation and area-level deprivation.*
